# Array comparative genomic hybridization of 18 pancreatic ductal adenocarcinomas and their autologous metastases

**DOI:** 10.1186/s13104-017-2886-0

**Published:** 2017-11-06

**Authors:** Valentin Rausch, Andreas Krieg, Jordi Camps, Bianca Behrens, Manfred Beier, Darawalee Wangsa, Kerstin Heselmeyer-Haddad, Stephan E. Baldus, Wolfram T. Knoefel, Thomas Ried, Nikolas H. Stoecklein

**Affiliations:** 1 Department of General, Visceral, and Pediatric Surgery, Heinrich-Heine-University and University Hospital Duesseldorf, Moorenstrasse 5, 40225 Duesseldorf, Germany; 2 Genetics Branch, Center for Cancer Research, National Cancer Institute, National Institutes of Health, Bethesda, MD USA; 3 Present Address: Gastrointestinal and Pancreatic Oncology Group, Institut d’Investigacions Biomèdiques August Pi i Sunyer (IDIBAPS), Centro de Investigación Biomédica en Red de Enfermedades Hepáticas y Digestivas (CIBEREHD), Hospital Clínic de Barcelona, Barcelona, Spain; 4 Institute of Human Genetics and Anthropology, Heinrich-Heine-University and University Hospital, Duesseldorf, Germany; 5 Department of Pathology, Heinrich-Heine-University and University Hospital, Duesseldorf, Germany; 6 Section of Cancer Genomics, Genetics Branch, Department of Health and Human Services, National Cancer Institute, National Institutes of Health, Center for Cancer Research, Bethesda, MD USA

**Keywords:** Intratumor hetereogeneity, Array-CGH, aCGH, Pancreatic cancer, PDAC, Pancreatic metastases

## Abstract

**Background:**

Mortality rates of pancreatic cancer remain high, which is mainly due to advanced disease and metastasis. We hypothesized that genomic copy number alterations are enriched in metastatic cells compared to autologous primary tumors, which may inform on cancer-related pathways possibly serving as potential targets for specific therapies. We investigated 18 pancreatic ductal adenocarcinomas, including 39 lymph node and 5 distant metastases after surgical resection. Analysis was performed with array-based comparative genomic hybridization (aCGH).

**Results:**

Metastases acquire a higher frequency of copy number alterations with the highest in distant metastasis (median = 42, lymph node metastases: median = 23, primary tumors: median = 17). In lymph node metastases, gains were prevalent on chromosome bands 8q11.23-q24.3, 12q14.1, 17p12.1, 21q22.12, and losses on 3p21.31, 4p14, 8p23.3-p11.21,17p12-11.2. Genes on amplified regions are involved in cancer-related pathways such as WNT-signaling, also involved in metastasis.

**Conclusions:**

Pancreatic cancers show a high degree of intratumor heterogeneity, which could lead to resistance of chemotherapy and worse outcome. ACGH analysis reveals regions preferentially gained or lost in synchronous metastases encoding for genes involved in cancer-related pathways, which could lead to novel therapeutic opportunities.

**Electronic supplementary material:**

The online version of this article (10.1186/s13104-017-2886-0) contains supplementary material, which is available to authorized users.

## Background

Pancreatic cancer is characterized by a poor prognosis making it the fourth leading cause for cancer related death in the US today [1]. Every year, about 46,000 patients in the US develop pancreatic cancer with ductal adenocarcinoma (PDAC) as the most common histological type. The 5-year survival rate of this disease is still significantly below 10% [1], which could not be improved during the last decades despite novel therapeutic strategies [1]. Surgery still remains the only curative option, although 5-year-survival in potentially resectable patients does not exceed 20% [2]. This poor prognosis could make PDAC the second leading cancer death before 2030 [3]. One reason for this poor prognosis is the lack of early specific symptoms; many patients therefore present at stages in which metastases have already occurred, which are highly resistant to conventional chemotherapy [4].

The overall pattern of genetic alterations in pancreatic cancer is well studied: long-known are specific alterations in primary lesions that are acquired through a well-defined time course to invasive carcinomas and are maintained in cell lines [5, 6]. Studies using whole genome sequencing have confirmed frequent mutations in prominent cancer associated genes, including *KRAS*, *TP53*, *SMAD4* and *CKDN2A* [7–10]. In addition to these common targets, there exists a landscape of heterogeneous alterations of low frequency that may contain druggable targets [11]. Those studies showed a high degree of intratumor heterogeneity (ITH), which translates to a significant diversity in molecular mechanisms involved in tumor progression. However, ITH between samples from primary tumors and their corresponding metastases of the same patient has only been studied recently: Yachida [12] and Campbell [13], using sequencing techniques showed a high grade of ITH between metastases and the primary tumor obtained from the same patient. One important mechanism underlying the heterogeneity in PDAC is thought to be telomere dysfunction [14], resulting in breakage-fusion-bridge cycles, leading to amplification or deletion of specific regions. Hence, ITH is explained by the rearrangement of an instable genome. Moreover, centrosome amplifications were reported as a source of chromosomal instability and ITH [15]. The degree of chromosomal instability is also a clinical predictor of treatment response [16, 17].

In our study, we focused on differences in the genomic aberration profile of primary tumors and autologous and synchronous lymph node (LNM) or distant organ metastases (OM). We hypothesized that cells that spread from primary PDAC and infiltrate lymph nodes or distant organs require a certain aberration profile. Since the presence of lymph node metastases is a poor prognostic factor in this disease [18], some of those features should also be present in loco-regional lymph node metastases. We expected that analysis of the genetic profile of primary tumors and their autologous lymph node metastases might reveal differences in those samples and therefore provide a panel of candidate genes that could be involved in promoting cancer cell metastasis.

In order to address these questions, we performed array-based comparative genomic hybridization (aCGH) to compare copy number alterations (CNAs) of primary tumors and their autologous metastases on a whole-genome level. This technique has already been used in other types of cancer, such as breast [19–24] or colorectal cancer [25–27] to study differences between lymph node metastases and primary tumors. The identification of CNAs that specifically appear in metastasized tumor cells might not only lead to a better understanding of the genetic basis of PDAC metastasis but also unmask potential therapeutic targets that could provide a better efficacy against advanced disease, with the hope of improving the survival rates of PDAC patients.

## Methods

### Clinical samples

All patients who underwent surgery with curative intent for histologically confirmed PDAC between 2002 and 2009 at the Department for Surgery (A) at the University Hospital Duesseldorf were identified by medical chart review. The ethics committee of the Medical Faculty of the Heinrich-Heine-University Duesseldorf approved the investigation of FFPE material as performed in this investigation (vote Number 3821). Consent to participate was waived, since patient data were strictly pseudonymized and analysis had no detrimental effect on the participants. Inclusion criteria were: patients with microscopically confirmed metastases in more than one lymph node and PDAC at any localization within the pancreas, thus being classified at least as UICC stage IIb. All types of surgical resection were eligible. Exclusion criteria were as follows: other malignancies in the patient history, pancreatic tumors other than adenocarcinoma or adenocarcinoma of the Ampulla of Vater, neoadjuvant radio- or chemotherapy and very small lymph node metastases (< 0.3 cm). Resectability of the tumor in surgery was not considered in the selection of the cases, thus cases with R1-resection were also included in the study. Applying these selection criteria, a total number of 52 patients were eligible to be included into our study.

The primary tumor was localized in the pancreatic head in 16 cases, one tumor extended from the head to the body of the pancreas and one tumor involved the pancreatic tail. The pancreatic tail tumor was surgically treated by resection of the distal pancreas, spleen and transverse colon including the left colonic flexure. Ten patients underwent the classical Whipple procedure (Pancreaticoduodenectomy) and seven patients underwent pylorus-preserving pancreaticoduodenectomy (PPPD). In all surgical procedures a lymph node dissection was performed resulting in a median number of 23 lymph nodes (range 12–55). Pathological examination of resected lymph nodes revealed a median number of 7 LNM (range 3–41). Tumor-free resection margins were achieved in 11 patients. After quality control, 18 patients were randomly selected from whom formalin-fixed and paraffin-embedded (FFPE) tissue specimen of the respective primary tumors (PT) and a total number of 39 lymph node metastases (LNM) were available for further analysis. The median age of our cohort was 64 years (range 44–81 years) (Table 1). In 4 cases, a total number of 5 liver metastases were found intraoperatively. In these cases, metastases had not been discovered preoperatively. Distant metastases were defined as organ metastases (OM) other than the pancreas and were also included in our analysis.

**Table 1:** Clinical and pathological data of patients included in study

Patient (no.)	Age at procedure	LNM^a^ (no.)	LN^b^ total (no.)	OM^c^	T	N	M	Grad.	R	Stage	Localization	Procedure
2	54	10	18	1	4	1	1	2	0	IV	Head	Whipple
8	62	30	37	1	3	1	1	3	0	IV	Head	Whipple
19	76	7	35	0	3	1	0	3	0	IIb	Head	Whipple
24	44	7	19	1	3	1	1	3	1	IV	Head	PPPD
28	65	3	39	1	3	1	1	3	0	IV	Head	PPPD
33	67	6	27	1	3	1	1	3	1	IV	Head	PPPD
41	66	7	22	0	3	1	0	2	0	IIb	Head	Whipple
42	75	3	23	0	3	1	0	3	0	IIb	Head, body	Whipple
44	55	8	16	0	3	1	0	3	0	IIb	Head	PPPD
45	62	41	55	1	3	1	1	2	1	IV	Head	Whipple
47	81	7	12	0	3	1	0	3	1	IIb	Head	Whipple
48	64	9	32	0	4	1	0	3	1	III	Head	PPPD
49	79	17	50	1	3	1	1	3	1	IV	Head	Whipple
50	47	4	22	1	3	1	1	3	1	IV	Head	PPPD
51	59	4	24	1	3	1	0	3	0	IV	Head	PPPD
52	61	8	12	0	3	1	0	3	0	IIb	Head	Whipple
53	61	3	36	1	3	1	1	2	0	IV	Tail	Distal pancreatectomy
54	72	5	23	0	3	1	0	3	0	IIb	Head	Whipple

^a^Lymph node metastases

^b^Lymph nodes

^c^Distant metastases

### DNA extraction

**Fig. 1:**
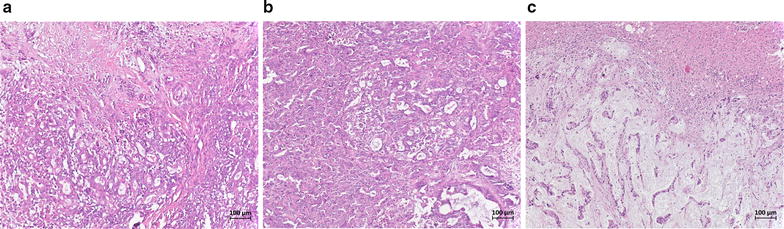
H&E stained samples from PDAC. **a** Sample section from a primary tumor (case 33). **b** Sample section from one of the lymph node metastasis (case 33). **c** Sample section from a distant metastasis (lung) (case 33)

Genomic DNA was isolated from FFPE tissue specimens of primary tumors, LNMs and OMs as recently described [52]. Therefore, hematoxylin and eosin (HE) stained sections of all specimens were reviewed by a pathologist (Baldus, SE) who marked representative tumor regions on the microscope slide (Fig. 1). Serial 10 µm thick sections were then processed from the respective FFPE blocks. Marked microscope slides served to localize the tumor region on the respective FFPE block. DNA extraction was performed using the Qiagen QIAmp DNA FFPE Tissue Kit (Qiagen, Hilden, Germany) according to manufacturer’s instructions. As reference DNA for aCGH analysis we extracted tissue from histologically confirmed normal lymph nodes of the corresponding patient to avoid copy number variations of non-pathological regions to interfere with our results. Purity and concentration of the DNA was measured spectrophotometrically and integrity tested on a 1% agarose gel.

### Array comparative genomic hybridization

Before array CGH was performed, DNA was RNase (RNase, DNase free, Roche, USA) treated and purified a second time before labeling using Kreatech’s (Kreatech Diagnostics, Amsterdam, Netherlands) non-enzymatic Universal Linkage System (ULS) according to the manufacturers’ instructions. We used reversed labeling for our samples: ULS-Cy3 to label sample-DNA and ULS-Cy5 to label the reference DNA. After purification of the labeled DNA and quality control with spectrophotometry, the DNA was analyzed with aCGH using 8 × 60 K arrays from Agilent’s SurePrint G3 Human CGH Microarray Kit (Agilent Technologies, Santa Clara, CA, USA). Hybridized slides were then washed and scanned by the Agilent SureScan Microarray Scanner System. Normalized, quality filtered log10-ratios were obtained using Agilent Feature Extraction software. Initial data visualization was performed using Agilent Genomic Workbench (version 6.5.0.18).

### Data analysis

Further bioinformatic analysis was accomplished using an R-pipeline [53] composed by several R add-ons available from Bioconductor (bioconductor.org) [54] and CRAN (cran.r-project.org) projects. Prior to this analysis, the log10 ratios from the Agilent Feature Extraction software were converted to log2 ratios. Improper oligonucleotides were removed in quality control using the same flags as in the Agilent Feature Extraction tool: oligonucleotides were removed if gIsSaturated = 1 or rIsSaturated = 1 to exclude over- or undersaturated spots, gIsFeatNonUnifOL = 1 or rIsFeatNonUnifOL = 1 or Log Ratio = 0 to exclude spots that exhibited implausible information, meaning intensities of individual pixels on one spot were not homogeneous enough or log ratio was set to 0 if the background exceeded the signal intensity.

For breakpoint detection we used the “lawsglad”-algorithm [55] from the GLAD-function [56]. We applied the default parameters, except median center = FALSE, since centering of the arrays is carried out before analyzing the data in our pipeline. Only calls of aberrant regions spanning at least three oligos and with a minimum absolute log2-ratio of 0.2 were considered for further analysis. Hence, increases in sample to reference-ratio > 1.2 were defined as gains, decreases in sample to reference ratio < 0.8 were defined as losses. Since our main focus was the general differentiation of altered regions between tumor cells from different sites, no further distinction was made for high-level amplification or homozygous losses. Overviews of the genome were generated from this data with a modified plot-function of the aCGH-database. Shared aberrations were defined as any region in two or more samples that was deleted or amplified. Private aberrations were defined as those only present in one sample. For visualization of the data we used a modified plot function of the “aCGH”-package from Bioconductor project.

Statistical differences in the number of CNA were assessed using nonparametric testing (Mann–Whitney U test). A p < 0.05 was defined as statistical significance.

Enriched genetic alterations in metastatic cells were found by comparing the appearance of each CNA in primary tumors and corresponding lymph node metastases or distant metastases. Enrichment was defined as significant differences in frequencies of CNA in aCGH-data of two corresponding samples. Significance was assessed with a standard Fishers exact test for 3 × 2 contingency tables (gain, loss, no change in two samples). Significantly enriched (p ≤ 0.05) alterations present in > 25% of respective the samples were then analyzed with Panther classification system (www.pantherdb.org) [57].

For calculation of the genetic distance of the samples, aberrations were classified as shared (present in more than one sample) or private (distinct alterations in one sample). Asymmetric binary distance between the samples was then calculated and plotted in a matrix. The binary distance was calculated by dividing all private regions in two arrays through all segmented regions; this is represented as a non-dimensional number between 0.0 and 1.0. Hence, higher values indicate a larger binary distance and therefore a higher level of heterogeneity between two distinct samples. The matrix of the binary distance data was used to calculate the mean distance between two samples of several subsets of samples. Differences in mean distances were tested for statistical significance using a Mann–Whitney-U test.

## Results

### CNAs of primary tumors and metastases

**Fig. 2:**
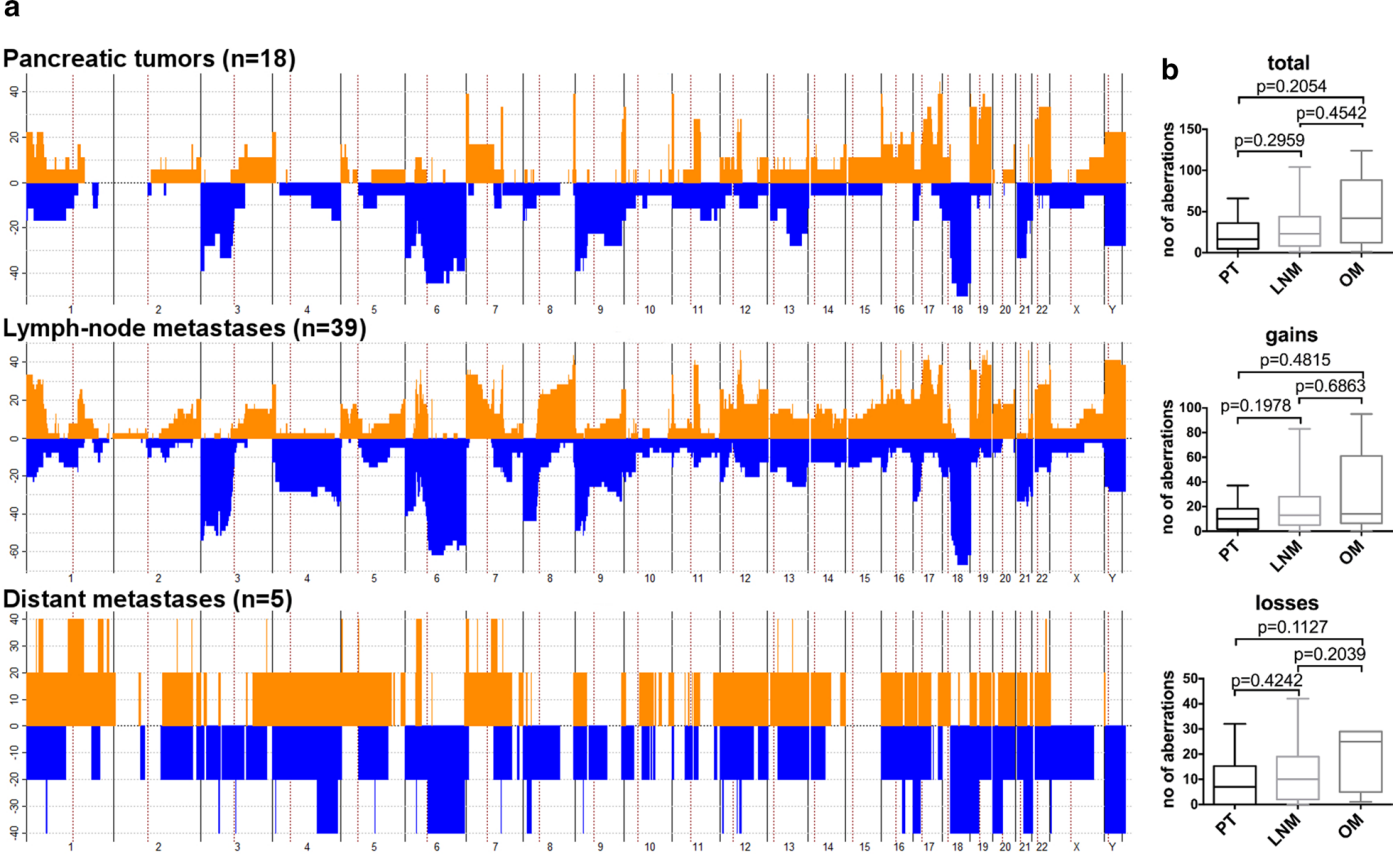
**a** Penetrance plot of genomic alterations depending on localization. **b** Total number of median gains and losses in primary tumors (PT), lymph node metastases (LNM) and distant metastases (OM)

Profiles from the aCGH-data displayed copy number alterations (CNAs) in all primary tumors and metastases (Fig. 2a, b, see also Additional file 1: Figure S1A–C). However, one sample (case45_PT1) exhibited alterations with less than three oligonucleotides and was therefore excluded from further analysis.

CNAs were defined as gains (more than 1.2 in sample to reference ratio) or losses (less than 0.8 in sample to reference ratio). Although not statistically significant, distant organ metastases (OM, n = 5) displayed the highest number of CNAs (median CNAs: 42, 1–124), followed by lymph node metastases (LNM, n = 39; median CNAs: 23, 1–104) and primary tumors (PT, n = 18; median CNAs: 17, 0-66). In addition, we observed a tendency toward a higher frequency of gains in the primary tumors with a median of 13 gained regions (0-95) compared to a median of 9.5 losses (0–42) (p = 0.0709).

The regions most commonly gained in primary tumors mapped to chromosome bands 17q25.1 (44.4%), 7p22.3 (38.9%), 8q24.3 (38.9%), 11p15.5 (38.9%), 16p13.3 (38.9%), 17q24.3-q25.3 (38.9%), 19p13.3 (38.9%), 19q13.12-q13.2 (38.9%). The most commonly lost regions were 18q12.3-q22.3 (50%), 6p11.2–q13 (44.4%), 6q13-q22.31 (44.4%), 18q12.1-q12.3 (44.4%), 18q22.3-q23 (44.4%).

**Fig. 3:**
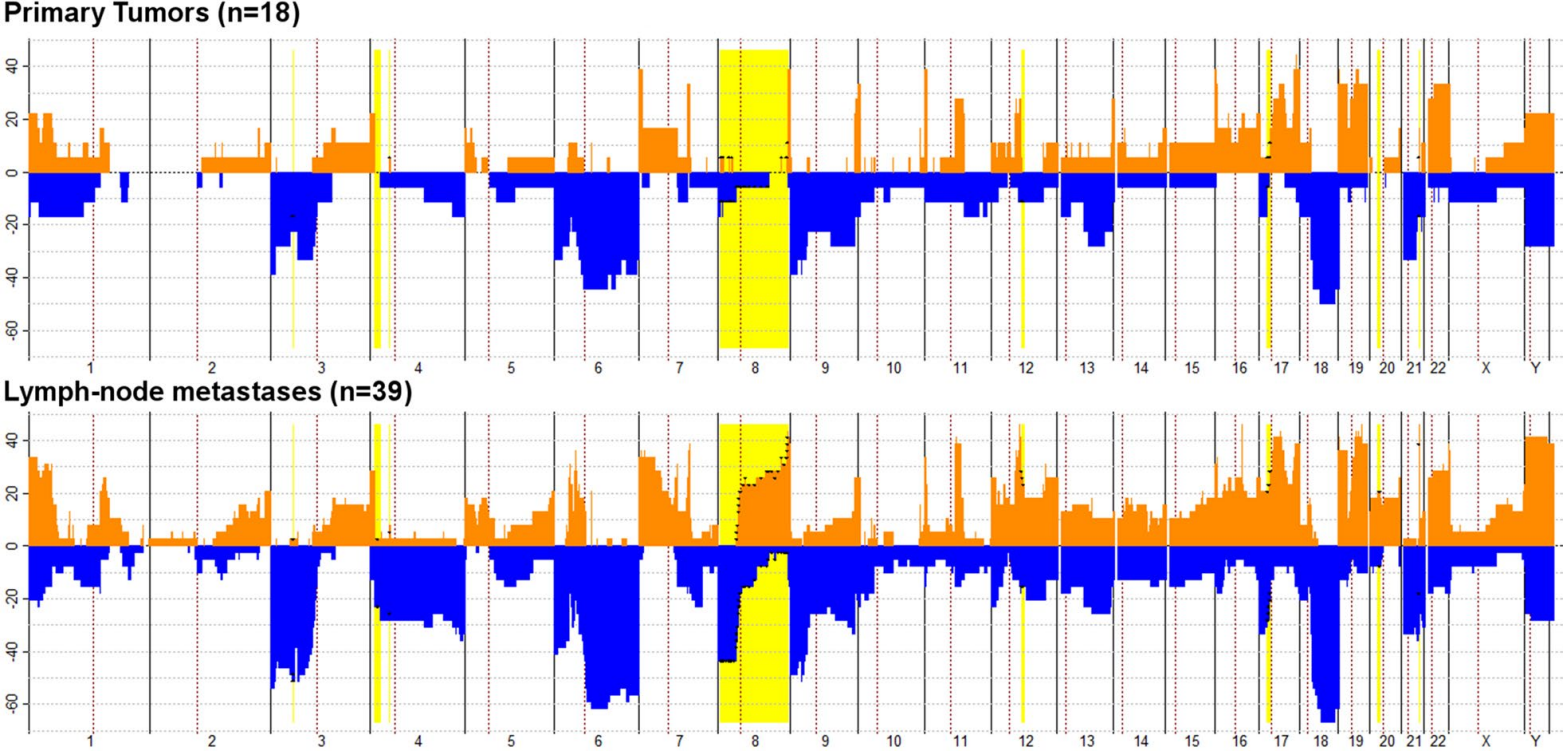
Penetrance plots of primary tumors (PT), and lymph node metastases (LNM). Yellow lines show significant enriched alterations in LNM

Next, we compared differences in CNAs between primary tumors, LNM and OM that were enriched in more than 25% of the samples (Additional file 2: Table S1A–H), since enrichment of specific regions could point to selected cancer-related genes. When comparing enriched gains between primary tumors and lymph node metastases, specific gains for lymph node metastases became evident on chromosome bands 8q11.23-q24.3, 12q14.1, 17p12.1 and 21q22.12. In addition, losses that could be observed only in lymph node metastases were on 3p21.31, 4p14, 8p23.3-p11.21 and 17p12-11.2 (Fig. 3). In contrast, distant metastases exhibited enriched gains on 1q32.1-q42.2 and 5q11.1, as well as enriched losses at 4q28.1-q32.3, 16q21, 17p11.2 and 20p13-q11.21 that could not be detected in matched primary tumors. Whereas enriched losses on chromosome 3p26.1-25.3 and 18q21.1-23 were specific for LNM when compared with OM, losses on chromosome 1p13.1-12 and 1q32.1-q41 and gains on chromosome 20p11.1-p11.21 were specifically present in OM. To get insight whether enriched genes in LNMs compared to PTs were involved in known cancer-related pathways, we performed gene ontology analysis on enriched regions using the Panther server (Additional file 2: Table S2). In total, 38 pathways were affected in enriched gains of LNM, 42 pathways were affected in enriched losses of LNM. Pathways most commonly affected with more than 2 genes in enriched losses of LNM were: FGF signaling, Apoptosis signaling, Angiogenesis, p53 signaling, Wnt signaling, and Gonadotropin releasing hormone receptor signaling. Only the Wnt-signaling pathway was affected with more than 2 genes in enriched gains of LNM.

### Comparison between CNAs of primary tumors and their matched metastases

**Fig. 4:**
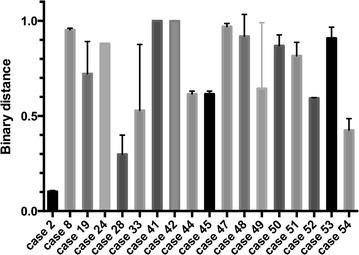
Mean binary distance and range of each primary tumor to their corresponding lymph node metastases

Since we noted differences in the number of aberrations and the aberration profiles between primary tumors and their metastases, we investigated samples for their shared genetic aberrations in order to better describe heterogeneity of the samples. To quantify heterogeneity, we measured the mean binary distance between different subsets (PT vs. corresponding LNM, between LNM throughout cases) as described above: the mean binary distance (on a scale ranging from 0 to 1, whereas 1 = no shared alterations, 0 = all alterations shared) between primary tumors and their corresponding lymph node metastases (Fig. 4) showed a significant higher (p = 0.0128) degree of heterogeneity (0.74, range 0.10–1.0) than between different lymph node metastases throughout all cases (0.87, range 0.07–1.0). Mean heterogeneity between LNM and their corresponding OM was also high (binary distance 0.77, range 0.32–1).

In individual cases we could find both, examples for highly heterogeneous and only slightly heterogeneous tumors: e.g. binary distance could be shown to spread widely in case 49, distance between LNM and corresponding OM ranged between 0.96 and 1.0, indicating a large heterogeneity between all samples from this case, whereas distance in case 24 ranged between 0.32 and 0.35, indicating only a small grade of heterogeneity.

## Discussion

Here we compared copy number gains and losses of primary PDAC tumors with their matched LNM and OM using aCGH. The overall CNA pattern of all samples was typical for PDAC [28–31]. However, while the aCGH data clearly showed that genetic heterogeneity between individual cases was higher than between different samples of an individual case, the CNA patterns of metastases diverged to some extent from their matched primary tumors. ITH, as indicated by heterogeneity between metastases and primary tumors from the same case is high in our cases, with a mean heterogeneity index of 0.74. Among metastases, gains on chromosome bands 8q24, 12q13, 16q12, 19q13, 21q22, 17q21 and losses at 18q and 6q13 were the most common aberrations. Most frequently enriched was gain of chromosome 8q (p < 0.043), comprising important cancer related genes such as *MYC* or *FZD6*. These genes are associated with an upregulation of Wnt-signaling, which is known to be altered in PDAC [11]. In addition to its role as potent oncogenic driver, *MYC* [32–36] contributes to chromosomal instability [37], which again can drive ITH. Gain of chromosome 8q has moreover been described in other cancers: in oral squamous cell cancer gain of 8q could be found to be associated with lymph-node metastasis [38], in colorectal cancer this alteration occurred significantly more frequent in corresponding brain metastases [39] and in breast cancer gain of MYC defines the transition from pre-invasive ductal carcinoma to invasive ductal carcinoma [40]. In view of these data, the involved gained 8q genes might be interesting candidates for metastatic drivers in PDAC. Gene ontology analysis of involved pathways hinted that several pathways could possibly be affected by gained or lost regions in lymph node metastases compared to primary tumors. Genes such as MYC play an important role in pathways relevant for cancer development such as cell-cycle control through Wnt-signaling. However, gene ontology analysis does not allow to differentiate between ‘driver-‘and ‘passenger-‘mutations and could therefore only be used to screen for candidates that could be studied in future studies.

Clearly, technical issues of the aCGH-methodology could also contribute to our observed differences. Despite our use of manual micro-dissection, the overall smaller number of alterations in primary tumors could result from the limitations of using bulk DNA for aCGH, leading to an average profile of the genetic alterations from several subclones. Moreover, only small regions of the primary tumor were extracted for aCGH analysis, hence not the entirety of clones is reflected with this method. Also, especially in PDAC with its strong desmoplastic stromal response, the amount of analyzed non-malignant cells can be significant.

Genetic heterogeneity between primary PDAC and their distant metastases as shown in our study could, however, also be observed on the sequence level using next generation sequencing (NGS): Yachida et al. [12] analyzed somatic mutations after extracting samples from different metastatic locations in seven PDAC patients and could detect the majority (mean 64%) of mutations in all samples from a patient. However, in two patients they collected samples from multiple sites of the sectioned primary tumor and found that subclones present in the primary tumor already reflected heterogeneity that was observed in the metastases, suggesting tumor spread to occur late in the course of the disease. In contrast, the study by Campbell et al. [13] who also sequenced samples from primary tumors and metastases of pancreatic cancer observed alterations, including driver mutations, that were exclusive in metastases, suggesting an earlier time point of dissemination followed by parallel clonal evolution of metastatic clones. These findings were possibly corroborated by data from experiments with a transgenic PDAC mouse model demonstrating that dissemination of cancer cells can be an early event [41], even occurring at the stage of pre-neoplastic lesions. In our study, we observed cases in which metastases were genetically close to their primary tumors but also more distant examples. Most probably, both scenarios can occur in PDAC patients explaining the observed different CNA patterns between primary tumors and their metastases: (a) cancer cells that leave the primary tumor (early) undergo additional changes due to clonal selection and adaptation at the metastatic site and (b) small subpopulations evolved within the primary tumor with the propensity to metastasize become the dominant metastatic clone. However, similarities between tumor samples from the same case might also be explained to some extent by other mechanisms, such as self-seeding. The hypothesis of self-seeding describes a mechanism in which metastases, once they infiltrated a distant organ, could seed themselves and re-enter their primary tumor [42].

Interestingly, ITH as a result of adaption processes can also be observed when sampling the same tumor over time [43–47]. Such genetic ITH enables more effective adaption to new microenvironments [48], e.g., at distant sites, and facilitates the development of therapeutic resistance [43, 49, 50]. In this context it is notable that increased levels of ITH are correlated to higher aggressiveness reflected by shorter survival in some cancer types [51]. It would be therefore highly interesting for future studies, to investigate, whether ITH in PDAC is a biomarker for aggressiveness and therapeutic resistance.

## Conclusions

Our comparative study of matched primary and metastatic PDAC tissue showed different levels of ITH and revealed CNAs that were enriched in metastatic lesions. These organ specific alterations might facilitate the identification of metastatic drivers in subsequent future studies, which might be amenable for future therapeutic interventions.

## Additional files

**Additional file 1: Figure S1.** ACGH-Profiles of each sample. The log-2-ratio is displayed in the y-axis, the localization on the genome is displayed on the x-axis. Called gains are marked orange, called losses are marked blue.

**Additional file 2: Table S1.** Enriched Gains and Losses (percentage of altered samples, range and involved Genes). A: Enriched Gains in LNM vs. PT. B: Enriched Losses in LNM vs. PT. C: Enriched Gains in OM vs. PT. D: Enriched Losses in PT vs. OM. E: Enriched Losses in LNM vs. OM. F: Enriched Gains in OM vs. LNM. G: Enriched Losses in OM vs. LNM. **Table S2.** Involved pathways, genes and respective protein class in enriched alterations. A: Altered pathways in enriched Gains (LNM vs PT). B: Altered pathways in enriched Losses (LNM vs PT).

## Abbreviations

aCGH: array-comparative genomic hybridization
CNA: copy number alteration
FFPE: formalin-fixated, paraffin-embedded
ITH: intratumor heterogeneity
LNM: lymph-node metastasis
NGS: next-generation sequencing
OM: distant metastasis
PDAC: pancreatic ductal adenocarcinoma
PPPD: pylorus-preserving pancreaticoduodenectomy
PT: primary tumor
UICC: Union internationale contre le cancer
