# Supplementary figures and images for: Array comparative genomic hybridization of 18 pancreatic ductal adenocarcinomas and their autologous metastases
Source: BMC Res Notes. 2017 Nov 6;10:560. doi: 10.1186/s13104-017-2886-0 (PMC5674747; doi:10.1186/s13104-017-2886-0)

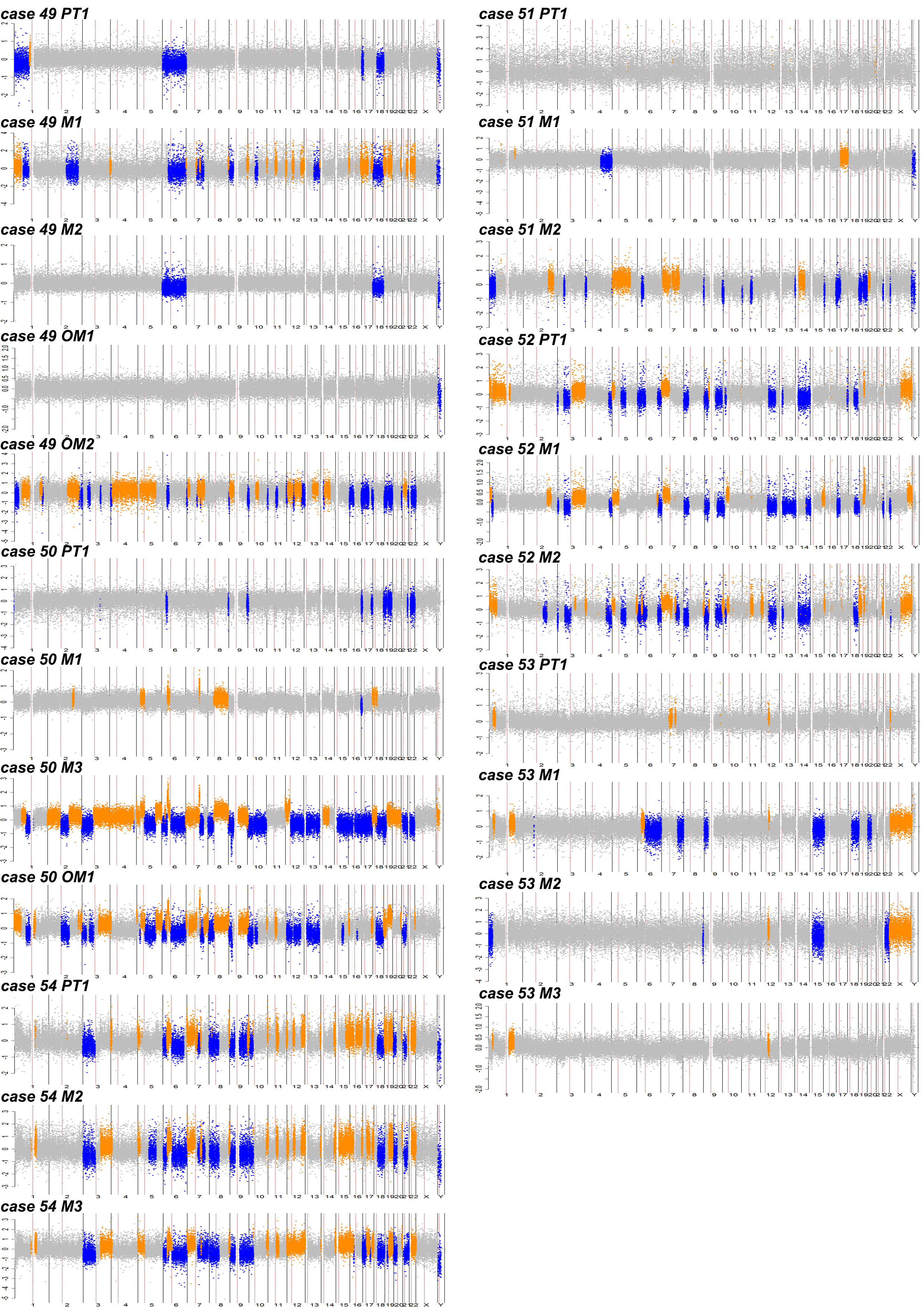

Supplement: Supplementary file 1 — Additional file 1: Figure S1. ACGH-Profiles of each sample. The log-2-ratio is displayed in the y-axis, the localization on the genome is displayed on the x-axis. Called gains are marked orange, called losses are marked blue. [file 13104_2017_2886_MOESM1_ESM.zip › SupplFig1c.png]

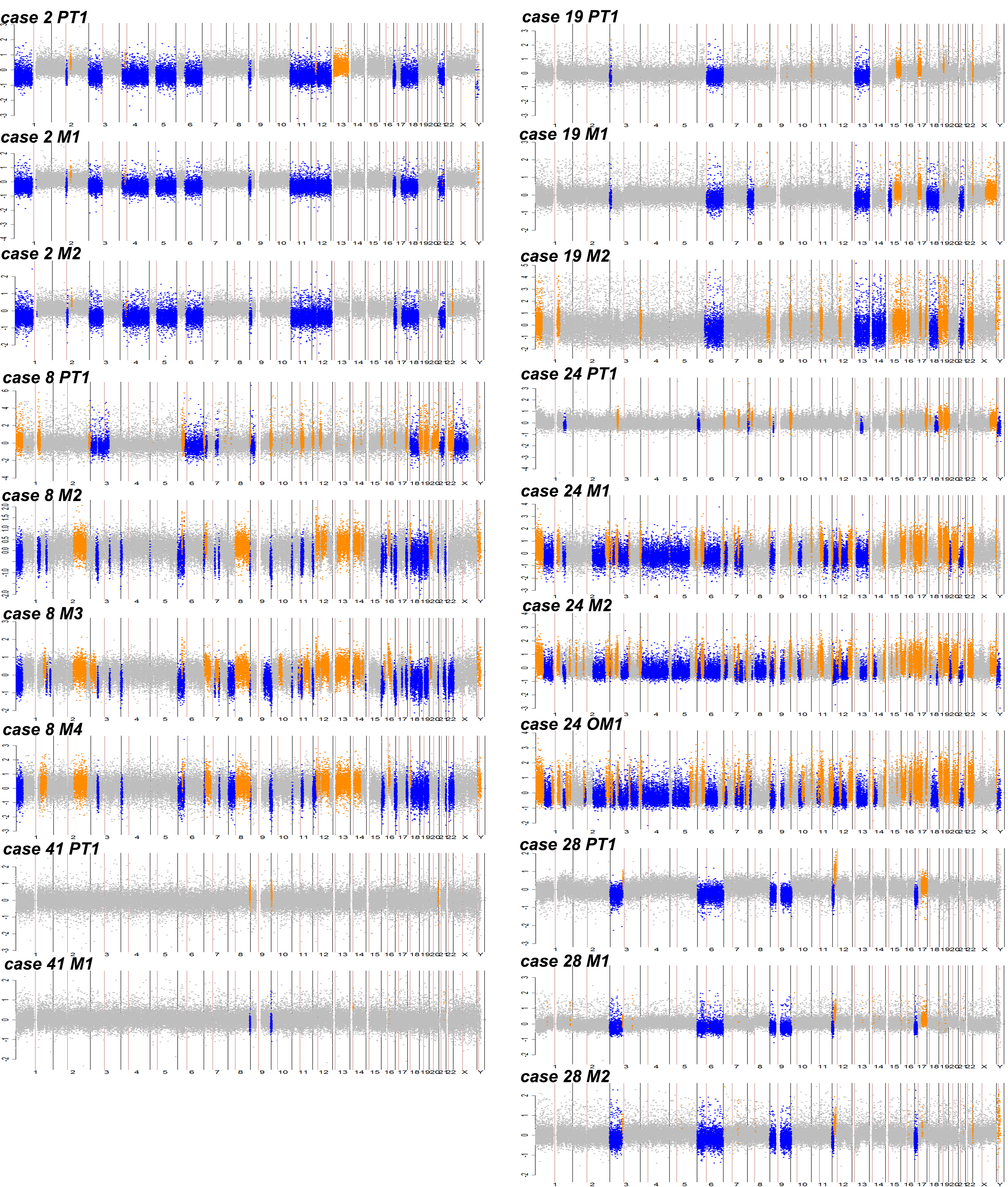

Supplement: Supplementary file 1 — Additional file 1: Figure S1. ACGH-Profiles of each sample. The log-2-ratio is displayed in the y-axis, the localization on the genome is displayed on the x-axis. Called gains are marked orange, called losses are marked blue. [file 13104_2017_2886_MOESM1_ESM.zip › SupplFig1a.png]

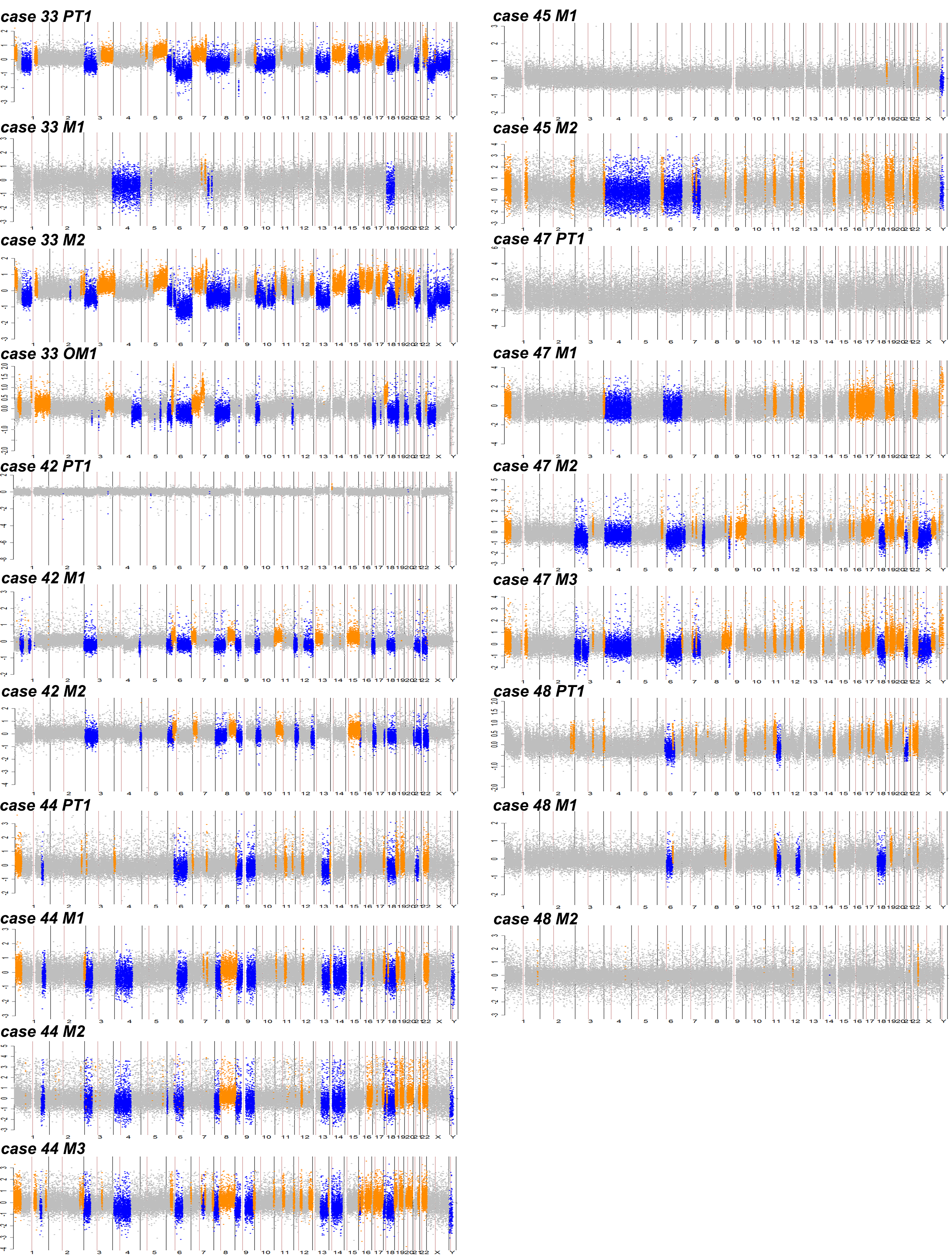

Supplement: Supplementary file 1 — Additional file 1: Figure S1. ACGH-Profiles of each sample. The log-2-ratio is displayed in the y-axis, the localization on the genome is displayed on the x-axis. Called gains are marked orange, called losses are marked blue. [file 13104_2017_2886_MOESM1_ESM.zip › SupplFig1b.png]
