# Supplementary material for: Array comparative genomic hybridization of 18 pancreatic ductal adenocarcinomas and their autologous metastases
Source: BMC Res Notes. 2017 Nov 6;10:560. doi: 10.1186/s13104-017-2886-0 (PMC5674747; doi:10.1186/s13104-017-2886-0)
Supplement: Supplementary file 2 — Additional file 2: Table S1. Enriched Gains and Losses (percentage of altered samples, range and involved Genes). A: Enriched Gains in LNM vs. PT. B: Enriched Losses in LNM vs. PT. C: Enriched Gains in OM vs. PT. D: Enriched Losses in PT vs. OM. E: Enriched Losses in LNM vs. OM. F: Enriched Gains in OM vs. LNM. G: Enriched Losses in OM vs. LNM. Table S2. Involved pathways, genes and respective protein class in enriched alterations. A: Altered pathways in enriched Gains (LNM vs PT). B: Altered pathways in enriched Losses (LNM vs PT). [file 13104_2017_2886_MOESM2_ESM.docx]

**SUPPLEMENTARY TABLE 1A:** Enriched Gains in LNM vs PT

| Chrom | Band | Range | Gains in Con-trol Group | % of Gains | pValue | Genes |
| --- | --- | --- | --- | --- | --- | --- |
| 8 | q11.23 — q12.1 | 55351769.5-55937767.5 | 10 | 25.64 | 0.012 | SOX17, RP1 |
| 8 | q21.11 — q22.1 | 77900045-96891578 | 10 | 25.64 | 0.031 | PXMP3, PKIA, FAM164A, IL7, STMN2, HEY1, MRPS28, TPD52, PC-1, ZBTB10, ZNF704, PAG1, FABP5, PMP2, FABP9, FABP12, IMPA1, SLC10A5, ZFAND1, CHMP4C, SNX16, RALYL, LRRCC1, E2F5, CA3, CA13, CA1, CA2, REXO1L1, PSKH2, ATP6V0D2, SLC7A13, FAM82B, CPNE3, CNGB3, CNBD1, DCAF4L2, MMP16, RIPK2, OSGIN2, NBN, DECR1, CALB1, TMEM64, NECAB1, TMEM55A, OTUD6B, LRRC69, SLC26A7, RUNX1T1, MTG8, FAM92A1, RBM12B, TMEM67, PDP1, CDH17, GEM, RAD54B, FSBP, ESRP1, DPY19L4, INTS8, CCNE2, TP53INP1, PLEKHF2 |
| 8 | q22.1 — q22.2 | 96891578-101091408,5 | 11 | 28.21 | 0.025 | GDF6, UQCRB, MTERFD1, PTDSS1, SDC2, PGCP, TSPYL5, MTDH, LYRIC, MATN2, RPL30, SNORA72, POP1, NIPAL2, KCNS2, STK3, OSR2, VPS13B, COX6C, RGS22 |
| 8 | q22.2 — q22.3 | 101091408,5-102327670 | 11 | 28.21 | 0.02 | RGS22, FBXO43, SPAG1, RNF19A, Dorfin, ANKRD46, SNX31, PABPC1, YWHAZ, ZNF706 |
| 8 | q22.3 — q23.3 | 102327670-115205131 | 11 | 28.21 | 0.015 | FKSG17, GRHL2, RRM2B, p53R2, UBR5, ODF1, KLF10, AZIN1, ATP6V1C1, BAALC, FZD6, Hfz6, CTHRC1, SLC25A32, DCAF13, WDSOF1, RIMS2, TM7SF4, DPYS, LRP12, ZFPM2, OXR1, ABRA, ANGPT1, RSPO2, EIF3E, EIF3S6, TTC35, TMEM74, TRHR, NUDCD1, ENY2, PKHD1L1, EBAG9, RCAS1, GOLSYN, AX748380, KCNV1, CSMD3 |
| 8 | q23.3 | 115205131-116107960 | 10 | 25.64 | 0.017 |  |
| 8 | q23.3 — q24.21 | 116107960-127984152 | 11 | 28.21 | 0.015 | EIF3H, UTP23, RAD21,SLC30A8, MED30, EXT1, SAMD12, TNFRSF11B, COLEC10, MAL2, NOV, ENPP2, TAF2, DSCC1, DEPDC6, COL14A1, MRPL13, MTBP, SNTB1, HAS2, HAS2AS, ZHX2, DERL1, WDR67, FAM83A, HCCC11, ZHX1, ATAD2, WDYHV1, FBXO32, KLHL38, ANXA13, FAM91A1, FER1L6, TMEM65, TRMT12, RNF139, TATDN1, NDUFB9, MTSS1, ZNF572, NSMCE2, TRIB1, FAM84B |
| 8 | q24.21 | 128113227.5-128440461.5 | 13 | 33.33 | 0.029 | POU5F1P1 |
| 8 | q24.21 — q24.23 | 128593489.5-139124113.5 | 12 | 30.77 | 0.007 | MYC, TMEM75, CCDC26, GSDMC, FAM49B, ASAP1, DDEF1IT1, ADCY8, EFR3A, OC90, HHLA1, KCNQ3, HPRG1, LRRC6, TMEM71, PHF20L1, TG, SLA, WISP1, NDRG1, Cap43, TARG1, ST3GAL1, ZFAT, KIAA1485, ZFATAS, KHDRBS3 |
| 8 | q24.23 | 139314964.5-139359304.5 | 13 | 33.33 | 0.029 | FAM135B |
| 8 | q24.23 | 139359304,5-139783966 | 14 | 35.90 | 0.029 | FAM135B |
| 8 | q24.23 — q24.3 | 139783966-140362716,5 | 15 | 38.46 | 0.014 | COL22A1 |
| 8 | q21.11 — q22.1 | 77900045-96891578 | 10 | 25.64 | 0.031 | PXMP3, PKIA, FAM164A, IL7, STMN2, HEY1, MRPS28, TPD52, PC-1, ZBTB10, ZNF704, PAG1, FABP5, PMP2, FABP9, FABP12, IMPA1, SLC10A5, ZFAND1, CHMP4C, SNX16, RALYL, LRRCC1, E2F5, CA3, CA13, CA1, CA2, REXO1L1, PSKH2, ATP6V0D2, SLC7A13, FAM82B, CPNE3, CNGB3, CNBD1, DCAF4L2, MMP16, RIPK2, OSGIN2, NBN, DECR1, CALB1, TMEM64, NECAB1, TMEM55A, OTUD6B, LRRC69, SLC26A7, RUNX1T1, MTG8, FAM92A1, RBM12B, TMEM67, PDP1, CDH17, GEM, RAD54B, FSBP, ESRP1, DPY19L4, INTS8, CCNE2, TP53INP1, PLEKHF2 |
| 8 | q24.3 | 140362716,5-140529080 | 15 | 38.46 | 0.043 |  |
| 8 | q24.3 | 140529080-140734665,5 | 16 | 41.03 | 0.04 | KCNK9 |
| 12 | q14.1 | 60336177.5-60747303.5 | 11 | 28.21 | 0.015 |  |
| 12 | q14.1 | 60747303,5-62595734 | 10 | 25.64 | 0.037 | FAM19A2 |
| 17 | p11.2 | 21473804,5-21657791 | 11 | 28.21 | 0.004 |  |
| 17 | p11.2 | 21657791-21873657 | 11 | 28.21 | 0.014 | FAM27L |
| 17 | p11.2 | 21873657-22006089,5 | 11 | 28.21 | 0.02 |  |

**SUPPLEMENTARY TABLE 1B:** Enriched Losses in LNM vs PT

| Chrom | Band | Range | Losses in Con-trol Group | % of Losses | pValue | Genes |
| --- | --- | --- | --- | --- | --- | --- |
| 3 | p21.31 | 44404945-44614829,5 | -20 | 51.28 | 0.024 | ZFP |
| 3 | p21.31 | 44614829.5-44767114.5 | -19 | 48.72 | 0.026 |  |
| 3 | p21.31 | 44767114,5-45851843 | -20 | 51.28 | 0.024 | KIAA1143, KIF15, TMEM42, TGM4, ZDHHC3, EXOSC7, CLEC3B, CDCP1, TMEM158, LARS2, LIMD1, SACM1L, SLC6A20 |
| 4 | p14 | 39833222,5-39876120 | -10 | 25.64 | 0.039 | PDS5A |
| 8 | p23.2 — p23.1 | 5124853-8288690 | -17 | 43.59 | 0.014 | MCPH1, ANGPT2, AGPAT5, LPAAT-e, XKR5, DEFB1, DEFA5, DEFB109, DEFB103B, SPAG11B, DEFB104A, DEFB106A, DEFB105A, DEFB107B, FAM90A7, SPAG11A, HE2, DEFB103A, DEFB4, FAM66E, PRAGMIN |
| 8 | p23.1 | 9225833.5-11769424.5 | -17 | 43.59 | 0.014 | TNKS, MSRA, UNQ9391, RP1L1, SOX7, PINX1, XKR6, MTMR9, AMAC1L2, TDH, FAM167A, BLK, GATA4, NEIL2, FDFT1, CTSB |
| 8 | p23.1 — p21.3 | 11769424,5-21892674 | -17 | 43.59 | 0.018 | DEFB136, DEFB135, DEFB134, DEFB130, ZNF705D, FAM66D, USP17L2, FAM86B1, FAM86B2, FAM66A, DEFB109, LONRF1, DLC1, TUSC3, HRF2, MSR1, SR-A, FGF20, EFHA2, ZDHHC2, CNOT7, VPS37A, MTMR7, SLC7A2, PDGFRL, MTUS1, FGL1, LFIRE1, PCM1, ASAH1, NAT1, NAT2, PSD3, SH2D4A, ChGn, CSGALNACT1, INTS10, LPL, SLC18A1, LZTS1, GFRA2, DOK2, XPO7, NPM2 |
| 8 | p21.3 | 21892674-22107335 | -17 | 43.59 | 0.014 | NPM2, FGF17, EPB49, FAM160B2, NUDT18, HR, hairless, REEP4, LGI3, SFTPC, BMP1, PHYHIP, POLR3D |
| 8 | p21.3 — p21.2 | 22107335-26606169 | -17 | 43.59 | 0.018 | PIWIL2, SLC39A14, PPP3CC, SORBS3, PDLIM2, BIN3, FLJ14107, EGR3, PEBP4, RHOBTB2, TNFRSF10B, MGC31957, TNFRSF10C, TNFRSF10D, TNFRSF10A, CHMP7, R3HCC1, LOXL2, ENTPD4, HT015, NKX3-1, NKX3A, NKX2-6, STC1, ADAM28, ADAMDEC1, ADAM7, NEFM, NEFL, DOCK5, PPP2R2A, GNRH1, KCTD9, CDCA2, EBF2, NIX, BNIP3L, PNMA2, DPYSL2, ADRA1A |
| 8 | p21.2 — p12 | 26606169-30678565 | -17 | 43.59 | 0.014 | ADRA1A, RB3, STMN4, TRIM35, PTK2B, CHRNA2, EPHX2, CLU, SCARA3, CCDC25, ESCO2, PBK, SCARA5, ELP3, PNOC, ZNF395, FBXO16, FZD3, INTS9, RC74, HMBOX1, KIF13B, DUSP4, TMEM66, foap-7, MBOAT4, DCTN6, RBPMS, GTF2E2, GSR, UBXN8 |
| 8 | p12 | 30678565-35597435 | -17 | 43.59 | 0.018 | TEX15, PURG, WRN, NRG1, FUT10, MAK16, RNF122, DUSP26, UNC5D |
| 8 | p11.23 | 36943201-37611965,5 | -15 | 38.46 | 0.011 | ZNF703, ERLIN2 |
| 8 | p11.23 | 37611965,5-37936139 | -14 | 35.90 | 0.029 | PROSC, BRF2, RAB11FIP1, GOT1L1, ADRB3, EIF4EBP1 |
| 8 | p11.23 | 37936139-37982498,5 | -13 | 33.33 | 0.027 | ASH2L |
| 8 | p11.23 — p11.21 | 37982498,5-41143422 | -12 | 30.77 | 0.041 | LSM1, CaSm, BAG4, DDHD2, PPAPDC1B, WHSC1L1, LETM2, FGFR1, MST123, RNF5, TACC1, PLEKHA2, HTRA4, TM2D2, ADAM9, ADAM32, tMDC II, tMDC, ADAM18, ADAM2, INDO, IDO2, INDOL1, ZMAT4, SFRP1 |
| 8 | p11.21 | 41938473-41994239 | -10 | 25.64 | 0.039 |  |
| 17 | p12 — p11.2 | 14870853,5-18646233 | -11 | 28.21 | 0.025 | TEKT3, CDRT4, FAM18B2, CDRT1, TRIM16, ZNF286A, TBC1D26, ADORA2B, ZSWIM7, TTC19, NCOR1, PIGL, UBB, TRPV2, VRL, SNORD49B, SNORD49A, SNORD65, ZNF287, ZNF624, CCDC144A, FAM106A, TACI, MPRIP, PLD6, BHD, FLCN, COPS3, SGN3, NT5M, MED9, RASD1, RAI1, SMCR5, SREBF1, TOM1L2, LRRC48, ATPAF2, DRG2, MYO15A, ALKBH5, LLGL1, FLII, SMCR7, TOP3A, SMCR8, SHMT1, EVPLL, LGALS9C, tl132, CCDC144B, TBC1D28, ZNF286B, FKHRL1P2, TRIM16L |
| 17 | p11.2 | 18646233-18846105,5 | -10 | 25.64 | 0.004 | FBXW10, FAM18B, PRPSAP2 |

**SUPPLEMENTARY TABLE 1C:** Enriched Gains in OM vs PT

| Chrom | Band | Range | Gains in Con-trol Group | % of Gains | pValue | Genes |
| --- | --- | --- | --- | --- | --- | --- |
| 1 | q32.1 — q32.2 | 200835836-207247629,5 | 2 | 40 | 0.006 | GPR25, KIF21B, CACNA1S, TMEM9, IGFN1, PKP1, TNNT2, HNTN1, LAD1, TNNI1, PHLDA3, CSRP1, RPS10P7, NAV1, IPO9, SHISA4, LMOD1, TIMM17A, RNPEP, ELF3, GPR37L1, ARL8A, PTPN7, PTPRV, LGR6, PPP1R12B, SYT2, KDM5B, RABIF, KLHL12, ADIPOR1, PAQR1, CYB5R1, B5R.1, TMEM183B, TMEM183A, PPFIA4, ADORA1, A1, FMOD, MYBPH, CHI3L1, CHIT1, BTG2, OPTC, ATP2B4, U42379, SNORA77, LAX1, ZC3H11A, SNRPE, SOX13, ETNK2, KISS1, GOLT1A, PLEKHA6, PPP1R15B, PIK3C2B, MDM4, MDMX, LRRN2, NFASC, CNTN2, TMEM81, RBBP5, DSTYK, TMCC2, NUAK2, KLHDC8A, LEMD1, PCTK3, MFSD4, SLC45A3, RAB7L1, SLC41A1, PM20D1, SLC26A9, FAM72A, AVPR1B, CTSE, NRG2, SRGAP2, IKBKE, RASSF5, LGTN, HCA56, DYRK3, RED, IL10, IL19, IL20, IL24, FAIM3, TOSO, PIGR, FCAMR, YOD1, PFKFB2 |
| 1 | q32.2 — q41 | 207247629.5-214785617.5 | 2 | 40 | 0.04 | C4BPB, CD55, DAF, CR2, CR1, CR1L, CD46, CD34, PLXNA2, CAMK1G, LAMB3, HSD11B1, TRAF3IP3, IRF6, SYT14, SERTAD4, HHAT, KCNH1, PX19, RCOR3, TRAF5, RD3, SLC30A1, NEK2, INTS7, DTL, PPP2R5A, SNORA16B, TMEM206, NENF, WA1, ATF3, FAM71A, BATF3, NSL1, TATDN3, LQK1, VASH2, ANGEL2, RPS6KC1, RPK118, PROX1, SMYD2, PTPN14, CENPF, FAM108A6 |
| 1 | q42.12 — q42.2 | 225992013.5-231340035.5 | 2 | 40 | 0.04 | EPHX1, TMEM63A, LEFTY1, P5CR2, PYCR2, LEFTY2, H3F3B, ACBD3, MIXL1, LIN9, PARP1, ITPKB, PSEN2, CABC1, CDC42BPA, ZNF678, JMJD4, SNAP47, PRSS38, WNT9A, ARF1, MRPL55, GUK1, GJC2, OBSCN, TRIM11, TRIM17, HIST3H3, HIST3H2A, HIST3H2BB, RNF187, RHOU, RAB4A, SPHAR, NUP133, ABCB10, TAF5L, URB2, GALNT2, PGBD5, COG2, AGT, CAPN9, TTC13, ARV1, FAM89A, TRIM67 |
| 5 | q11.1 | 50638150.5-50689187.5 | 2 | 40 | 0.04 | CR604031, ISL1 |

**SUPPLEMENTARY TABLE 1D:** Enriched Losses in PT vs OM

| Chrom | Band | Range | Losses in Con-trol Group | % of Losses | pValue | Genes |
| --- | --- | --- | --- | --- | --- | --- |
| 4 | q28.1 — q32.3 | 126323705-167698361 | -2 | 40 | 0.048 | FAT4, INTU, SLC25A31, HSPA4L, PLK4, MFSD8, LARP1B, PGRMC2, PHF17, SCLT1, PABPC4L, PCDH18, SLC7A11, CCRN4L, ELF2, NDUFC1, NARG1, RAB33B, SETD7, MGST2, MAML3, SCOC, CLGN, ELMOD2, UCP1, TBC1D9, RNF150, ZNF330, IL15, IL-15, INPP4B, USP38, GAB1, SMARCA5, GYPE, BC029578, GYPB, GYPA, HHIP, ANAPC10, ABCE1, OTUD4, SMAD1, MMAA, C4orf51, ZNF827, lsm6, SLC10A7, POU4F2, TTC29, EDNRA, ETA, TMEM184C, PRMT10, ARHGAP10, graf-2, NR3C2, MR, DCLK2, LRBA, MAB21L2, RPS3A, SNORD73A, SH3D19, FAM160A1, PET112, FBXW7, TMEM154, TIGD4, ARFIP1, FHDC1, TRIM2, ANX2P2, MND1, TLR2, TIL4, RNF175, SFRP2, DCHS2, PLRG1, FGB, FGA, FGG, LRAT, RBM46, MGC27016, MAP9, GUCY1A3, GUCY1B3, GC-S-beta-1, ACCN5, TDO2, CTSO, PDGFC, GLRB, GRIA2, GRIA4, TMEM144, RXFP1, LGR7, ETFDH, PPID, FNIP2, RAPGEF2, CR595965, FSTL5, NAF1, NPY1R, NPY5R, TKTL2, MARCH1, TRIM61, TRIM60, TRIM75, TMEM192, KLHL2, GK, SC4MOL, CPE, TLL1, SPOCK3, N-Tes |
| 16 | q21 | 58769135-65737157 | -2 | 40 | 0.045 | CDH8, CDH11 |
| 17 | p11.2 | 18646233-19629716 | -2 | 40 | 0.021 | FBXW10, FAM18B, PRPSAP2, SLC5A10, FAM83G, GRAP, EPN2, B9D1, MAPK7, MFAP4, RNF112, SLC47A1, SNORA59B, ALDH3A2, SLC47A2 |
| 20 | p13 | 60761-4413347,5 | -2 | 40 | 0.021 | DEFB125, DEFB126, DEFB127, DEFB128, DEFB129, DEFB132, ZCCHC3, SOX12, NRSN2, TRIB3, RBCK1, TBC1D20, CSNK2A1, TCF15, SRXN1, SCRT2, FAM110A, F10, ANGPT4, RSPO4, PSMF1, RAD21L1, SDCBP2, SITAC18, FKBP12-Exip2, NSFL1C, SIRPB2, SIRPD, SIRPB1, SIRPG, PDYN, STK35, TGM3, TGM6, SNRPB, SNORD119, ZNF343, TMC2, NOP56, SNORD110, SNORA51, SNORD86, SNORD56, SNORD57, IDH3B, CPXM1, FAM113A, VPS16, PTPRA, GNRH2, MRPS26, OXT, AVP, FASTKD5, ProSAPiP1, DDRGK1, ITPA, SLC4A11, ATRN, GFRA4, ADAM33, SIGLEC1, HSPA12B, SPEF1, CENPB, CDC25B, MAVS, VISA, PANK2, RNF24, SMOX, ADRA1D |
| 20 | p13 | 4413347.5-4669522.5 | -2 | 40 | 0.04 |  |
| 20 | p13 — p12.1 | 4669522.5-17444991.5 | -2 | 40 | 0.04 | PRNP, PRND, M8, RASSF2, SLC23A2, PCNA, CDS2, PROKR2, RP5-1022P6.2, CHGB, TRMT6, MCM8, CRLS1, LRRN4, FERMT1, DTGCU2, BMP2, HAO1, TMX4, PLCB1, PLCB4, PAK7, ANKRD5, SNAP25, MKKS, JAG1, BTBD3, SPTLC3, ISM1, TASP1, ESF1, SEL1L2, MACROD2, FLRT3, KIF16B, SNRPB2, OTOR, PCSK2 |
| 20 | p12.1 — p11.1-q11.21 | 17444991.5-28038135.5 | -2 | 40 | 0.006 | PCSK2, BFSP1, DSTN, RRBP1, BANF2, SNX5, SNORD17, OVOL2, CSRP2BP, ZNF133, POLR3F, SEC23B, DTD1, HSPC072, SLC24A3, RIN2, NAT5, CRNKL1, INSM1, RALGAPA2, PLK1S1, XRN2, NKX2-4, NKX2-2, PAX1, FOXA2, SSTR4, THBD, CD93, AX747171, NXT1, GZF1, NAPB, CSTL1, CST11, CST8, CSTT, CST9L, CST9, CST3, CST4, CST1, CST2, CST5, GGTLC1, TMEM90B, CST7, ACSS1, VSX1, ENTPD6, CD39L2, PYGB, ABHD12, GINS1, NINL, NANP, ZNF337, FAM182B, FAM182A |

**SUPPLEMENTARY TABLE 1E:** Enriched Losses in LNM vs OM*

| Chrom | Band | Range | Losses in Con-trol Group | % of Losses | pValue | Genes |
| --- | --- | --- | --- | --- | --- | --- |
| 3 | p26.1 — p25.3 | 8299114-9306948,5 | -19 | 48.72 | 0.013 | LMCD1, CAV3, OXTR, RAD18, SRGAP3 |
| 3 | p25.3 | 9306948.5-10507498.5 | -18 | 46.15 | 0.022 | THUMPD3,SETD5, LHFPL4, MTMR14, CPNE9, BRPF1, OGG1, CAMK1, TADA3, ARPC4, ARC20, TTLL3, RPUSD3, CIDEC, CICE, IL17RE, IL17RC, CRELD1, PRRT3, TMEM111, FANCD2, VHL, IRAK2, TATDN2, GHRL, GHRLOS, SEC13, ATP2B2 |
| 3 | p25.3 | 10507498,5-10893953 | -17 | 43.59 | 0.044 | ATP2B2, SLC6A11 |
| 3 | p25.3 — p25.1 | 11298486-15559523,5 | -17 | 43.59 | 0.022 | HRH1, ATG7, VGLL4, SYN2, TIMP4, PPARG, TSEN2, MKRN2, RAF1, TMEM40, CAND2, RPL32, IQSEC1, NUP210, HDAC11, FBLN2, TPRXL, CHCHD4, TMEM43, XPC, SLC6A6, GRIP2, FGD5, NR2C2, MRPS25, ZFYVE20, DIVA, CAPN7, SH3BP5, METTL6, EAF1, COLQ |
| 18 | q21.1 | 43880883-46590878,5 | -26 | 66.67 | 0.02 | RNF165, LOXHD1, ST8SIA5, PIAS2, TCEB3CL, TCEB3C, TCEB3B, HDHD2, IER3IP1, SMAD2, ZBTB7C, SMAD7, DYM |
| 18 | q23 | 77049870-78029687 | -24 | 61.54 | 0.041 | ATP9B, NFATC1, CTDP1, KCNG2, PQLC1, HSBP1L1, TXNL4A, ADNP2, PARD6G |

* No significant enriched Gains in LNM vs OM

**SUPPLEMENTARY TABLE 1F:** Enriched Gains in OM vs LNM

| Chrom | Band | Range | Gains in Con-trol Group | % of Losses | pValue | Genes |
| --- | --- | --- | --- | --- | --- | --- |
| 1 | p13.1 — p12 | 117370979.5-118497708.5 | 2 | 40 | 0.012 | IGSF2, TTF2, TRIM45, VTCN1, MAN1A2, FAM46C, GDAP2, WDR3, SPAG17 |
| 1 | p12 | 118497708.5-119959378.5 | 2 | 40 | 0.044 | WDR3, SPAG17, WARS2, AK125759, BC063600, BC043601, HSD3B2 |
| 1 | q32.1 — q32.2 | 200835836-207247629,5 | 2 | 40 | 0.006 | GPR25, C1orf106, C1orf81, KIF21B, KIAA0449, BC016656, CACNA1S, TMEM9, IGFN1, PKP1, TNNT2, HNTN1, AK055533, LAD1, TNNI1, PHLDA3, CSRP1, AK055590, RPS10P7, AX747377, NAV1, IPO9, SHISA4, LMOD1, TIMM17A, RNPEP, ELF3, GPR37L1, ARL8A, PTPN7, PTPRV, LGR6, VTS20631, PPP1R12B, SYT2, KDM5B, LOC641515, AX721218, BC049825, LOC148709, RABIF, KLHL12, ADIPOR1, PAQR1, CYB5R1, B5R.1, TMEM183B, TMEM183A, C1orf37, CR591805, PPFIA4, KIAA0897, ADORA1, A1, FMOD, MYBPH, CHI3L1, AK095458, BC034684, FLJ00314, CHIT1, AK309664, AK024293, BTG2, OPTC, ATP2B4, U42379, SNORA77, LAX1, ZC3H11A, SNRPE, C1orf157, SOX13, ETNK2, HMFT1716, BC038769, KISS1, GOLT1A, PLEKHA6, KIAA0969, LOC127841, PPP1R15B, PIK3C2B, PIK3C2B variant protein, MDM4, MDMX, LRRN2, NFASC, KIAA0756, AX746920, CNTN2, TMEM81, RBBP5, DSTYK, TMCC2, NUAK2, KLHDC8A, AX746481, LEMD1, LEMP-1(a), DM004220, AK095633, PCTK3, LOC284578, MFSD4, DKFZp761N1114, AF321617, SLC45A3, DKFZp666D0110, RAB7L1, SLC41A1, AX748016, PM20D1, AK055746, SLC26A9, FAM72A, AVPR1B, C1orf186, CTSE, CatE, NRG2, SRGAP2, IKBKE, RASSF5, LGTN, HCA56, DYRK3, RED, IL10, IL19, IL20, IL24, FAIM3, TOSO, PIGR, FCAMR, C1orf116, pfkfb2, YOD1, PFKFB2 |
| 1 | q32.2 — q32.3 | 207247629,5-212012050 | 2 | 40 | 0.015 | pfkfb2, PFKFB2, C4BPB, AX229788, CD55, DAF, CR2, CR1, CR1L, CD46, EU154352, EU154351, LOC148696, AK123177, CD34, PLXNA2, LOC642587, DM004461, CAMK1G, LAMB3, HSD11B1, TRAF3IP3, C1orf74, IRF6, C1orf107, SYT14, sytXIV, C1orf133, SERTAD4, HHAT, KCNH1, PX19, RCOR3, KIAA1343, TRAF5, C1orf97, RD3, SLC30A1, CR605189, NEK2 |
| 1 | q32.3 | 212012050-212331984 | 2 | 40 | 0.021 | INTS7, DKFZp434B168, DTL, BC010168 |
| 1 | q32.3 — q41 | 212331984-214785617,5 | 2 | 40 | 0.011 | PPP2R5A, SNORA16B, TMEM206, NENF, WA1, CR624623, ATF3, FAM71A, BATF3, NSL1, TATDN3, C1orf227, LQK1, VASH2, FLJ12505, ANGEL2, RPS6KC1, RPK118, AK092251, PROX1, SMYD2, HSKM-B, PTPN14, LMAN2/AP3S1 fusion, CENPF, FAM108A6 |

**SUPPLEMENTARY TABLE 1G:** Enriched Losses in OM vs LNM

| Chrom | Band | Range | Losses in Con-trol Group | % of Losses | pValue | Genes |
| --- | --- | --- | --- | --- | --- | --- |
| 20 | p11.21 | 23935427-25436449 | -2 | 40 | 0.05 | GGTLC1, AK090900, TMEM90B, CST7, C20orf3, ACSS1, VSX1, LOC284798, AX747658, ENTPD6, CD39L2, PYGB, BC128043, ABHD12, GINS1, NINL |
| 20 | p11.21 — p11.1-q11.21 | 25436449-28038135,5 | -2 | 40 | 0.018 | NINL, NANP, ZNF337, FAM182B, AL096727, BC052952, LOC100134868, FAM182A, C20orf191, BC036544 |

**SUPPLEMENTARY TABLE 2A:** Pathways altered in Enriched Gains (LNM vs PT >2 genes)

| Pathway involved | Gene | Protein class |
| --- | --- | --- |
| 5HT1 type receptor mediated signaling pathway | KCNK9 | voltage-gated potassium channel, voltage-gated ion channel |
| 5HT2 type receptor mediated signaling pathway | KCNK9 | voltage-gated potassium channel, voltage-gated ion channel |
| 5HT3 type receptor mediated signaling pathway | KCNK9 | voltage-gated potassium channel, voltage-gated ion channel |
| 5HT4 type receptor mediated signaling pathway | KCNK9 | voltage-gated potassium channel, voltage-gated ion channel |
| Alzheimer disease-presenilin pathway | FZD6  MMP16 | signaling molecule, G-protein coupled receptor |
| Angiogenesis | ANGPT1 | signaling molecule |
| CCKR signaling map | MYC | basic helix-loop-helix transcription factor, nucleic acid binding |
| Cadherin signaling pathway | FZD6  CDH17 | signaling molecule, G-protein coupled receptor  cell junction protein, cadherin |
| Cell cycle | CCNE2 | kinase activator |
| De novo purine biosynthesis | RRM2B | reductase |
| De novo pyrimidine deoxyribonucleotide biosynthesis | RRM2B | reductase |
| Dopamine receptor mediated signaling pathway | OC90  KCNK9 | phospholipase, extracellular matrix protein  voltage-gated potassium channel, voltage-gated ion channel |
| EGF receptor signaling pathway | YWHAZ | chaperone |
| Endothelin signaling pathway | ADCY8 | adenylate cyclase, guanylate cyclase |
| FGF signaling pathway | YWHAZ | chaperone |
| GABA-B_receptor_II_signaling | ADCY8 | adenylate cyclase, guanylate cyclase |
| General transcription regulation | TAF2 | transcription factor |
| Hedgehog signaling pathway | UBR5 | ubiquitin-protein ligase |
| Heterotrimeric G-protein signaling pathway-Gi alpha and Gs alpha mediated pathway | ADCY8 | adenylate cyclase, guanylate cyclase |
| Heterotrimeric G-protein signaling pathway-rod outer segment phototransduction | CNGB3 | cyclic nucleotide-gated ion channel, voltage-gated potassium channel, voltage-gated ion channel, cyclic nucleotide-gated ion channel |
| Inflammation mediated by chemokine and cytokine signaling pathway | COL14A1 | Transporter, receptor |
| Integrin signalling pathway | COL14A1  ASAP1 | Transporter, receptor  nucleic acid binding, G-protein modulator |
| Interleukin signaling pathway | IL7  MYC | interleukin superfamily  basic helix-loop-helix transcription factor, nucleic acid binding |
| Nicotine pharmacodynamics pathway | OC90  KCNK9 | phospholipase, extracellular matrix protein  voltage-gated potassium channel, voltage-gated ion channel |
| Notch signaling pathway | HEY1 | basic helix-loop-helix transcription factor, nucleic acid binding |
| Opioid proenkephalin pathway | KCNK9 | voltage-gated potassium channel, voltage-gated ion channel |
| Opioid proopiomelanocortin pathway | KCNK9 | voltage-gated potassium channel, voltage-gated ion channel |
| Oxidative stress response | MYC | basic helix-loop-helix transcription factor, nucleic acid binding |
| PDGF signaling pathway | MYC | basic helix-loop-helix transcription factor, nucleic acid binding |
| PI3 kinase pathway | YWHAZ | chaperone |
| Parkinson disease | CCNE2  YWHAZ | kinase activator  chaperone |
| Pyrimidine Metabolism | DPYS | hydrolase |
| Synaptic_vesicle_trafficking | RIMS2 | G-protein modulator |
| TGF-beta signaling pathway | GDF6 | growth factor |
| Thyrotropin-releasing hormone receptor signaling pathway | TRHR | G-protein coupled receptor |
| Transcription regulation by bZIP transcription factor | TAF2 | transcription factor |
| Wnt signaling pathway | RAD54B  MYC  CDH17  FZD6 | basic helix-loop-helix transcription factor, nucleid acid binding  cell junction protein, cadherin  signaling molecule, G-protein coupled receptor |
| p53 pathway feedback loops 2 | MYC | basic helix-loop-helix transcription factor, nucleic acid binding |

**SUPPLEMENTARY TABLE 2B:** Pathways altered in Enriched Losses (LNM vs PT, >2 genes)

| Pathway involved | Gene | Protein class |
| --- | --- | --- |
| Adrenaline and noradrenaline biosynthesis | SLC6A20 | cation transporter |
| Alpha adrenergic receptor signaling pathway | ADRA1A | G-protein coupled receptor |
| Alzheimer disease-amyloid secretase pathway | ADAM9 |  |
| Alzheimer disease-presenilin pathway | FZD3 | signaling molecule, G-protein coupled receptor |
| Angiogenesis | FZD3 | signaling molecule, G-protein coupled receptor |
|  | SFRP1 | signaling molecule, G-protein coupled receptor |
|  | FGFR1 |  |
|  | ANGPT2 | signaling molecule |
|  | DOK2 |  |
| Apoptosis signaling pathway | TNFRSF10B | tumor necrosis factor receptor |
|  | BAG4 | chaperone |
|  | TNFRSF10C | tumor necrosis factor receptor |
|  | TNFRSF10A | tumor necrosis factor receptor |
|  | TNFRSF10D | tumor necrosis factor receptor |
| Asparagine and aspartate biosynthesis | GOT1L1 |  |
| Axon guidance mediated by netrin | UNC5D | receptor |
| Axon guidance mediated by semaphorins | DPYSL2 | hydrolase |
| B cell activation | PPP3CC  BLK | protein phosphatase  non-receptor tyrosine protein kinase |
| Beta3 adrenergic receptor signaling pathway | ADRB3 | G-protein coupled receptor |
| CCKR signaling map | CLU |  |
|  | EIF4EBP1 | translation factor |
|  | PTK2B | non-receptor tyrosine protein kinase |
| Cadherin signaling pathway | FZD3 | signaling molecule, G-protein coupled receptor |
| Cholesterol biosynthesis | FDFT1 |  |
| Cytoskeletal regulation by Rho GTPase | STMN4 |  |
| EGF receptor signaling pathway | NRG1  PEBP4 | growth factor |
| FGF signaling pathway | FGF17 | growth factor |
|  | PEBP4 |  |
|  | FGFR1 |  |
|  | FGF20 | growth factor |
|  | PPP2R2A | protein phosphatase |
| General transcription regulation | BRF2  GTF2E2 | transcription factor  transcription factor |
| Gonadotropin releasing hormone receptor pathway | GATA4 | zinc finger transcription factor, nuclease |
|  | GNRH1 | peptide hormone |
|  | PTK2B | non-receptor tyrosine protein kinase |
| Heterotrimeric G-protein signaling pathway-Gi alpha and Gs alpha mediated pathway | ADRB3 | G-protein coupled receptor |
| Huntington disease | LARS2  NCOR1 | aminoacyl-tRNA synthetase  transcription cofactor, nucleic acid binding |
| Inflammation mediated by chemokine and cytokine signaling pathway | PTK2B | non-receptor tyrosine protein kinase |
| Integrin signalling pathway | PTK2B | non-receptor tyrosine protein kinase |
|  | BLK | non-receptor tyrosine protein kinase |
|  | DPYSL2 | hydrolase |
| Serine glycine biosynthesis | SHMT1 | methyltransferase |
| T cell activation | PPP3CC | protein phosphatase |
| TGF-beta signaling pathway | BMP1 | growth factor |
| Toll receptor signaling pathway | LRRC48 | Receptor, extracellular matrix protein |
| Toll_pathway_drosophila | LRRC48 | Receptor, extracellular matrix protein |
| Transcription regulation by bZIP transcription factor | GTF2E2 | transcription factor |
| Wnt signaling pathway | PPP3CC | protein phosphatase |
|  | FZD3 | signaling molecule, G-protein coupled receptor |
|  | SFRP1 | signaling molecule, G-protein coupled receptor |
| p38 MAPK pathway | EIF4EBP1 | translation factor |
| p53 pathway by glucose deprivation | EIF4EBP1 | translation factor |
| p53 pathway | TNFRSF10B | tumor necrosis factor receptor |
|  | WRN | DNA helicase, helicase |
|  | TNFRSF10A | tumor necrosis factor receptor |
|  | TNFRSF10D | tumor necrosis factor receptor |
